# Further Delineation of Developmental Delay with Gastrointestinal, Cardiovascular, Genitourinary, and Skeletal Abnormalities Caused by *ZNF699* Gene Mutation

**DOI:** 10.3390/genes13020168

**Published:** 2022-01-18

**Authors:** Mateusz Biela, Malgorzata Rydzanicz, Agnieszka Jankowska, Agnieszka Szlagatys-Sidorkiewicz, Anna Rozensztrauch, Rafał Płoski, Robert Smigiel

**Affiliations:** 1 Department of Familial and Pediatric Nursing, Wroclaw Medical University, 51-618 Wroclaw, Poland; anna.rozensztrauch@umw.edu.pl (A.R.); robert.smigiel@umw.edu.pl (R.S.); 2 Department of Medical Genetics, Medical University of Warsaw, 02-106 Warsaw, Poland; rploski@wp.pl; 3 Department of Paediatrics, Gastroenterology, Allergology and Paediatric Nutrition, Medical University of Gdansk, 80-803 Gdansk, Poland; ajankowska@gumed.edu.pl (A.J.); agnieszka.szlagatys-sidorkiewicz@gumed.edu.pl (A.S.-S.)

**Keywords:** *ZNF699* gene, DEGCAGS syndrome, neurodevelopmental disorder

## Abstract

Until 2021, the *ZNF699* gene was not associated with any human genetic disease. There were only two studies exploring the associations between variants in *ZNF699* and alcohol dependence. In 2021 Bertoli-Avella et al. reported 13 patients with a *ZNF699* gene mutation. All patients presented global developmental delay and with systemic manifestations. A new phenotype was proposed and called DEGCAGS syndrome (OMIM 619488) (developmental delay with gastrointestinal, cardiovascular, genitourinary, and skeletal abnormalities). The DEGCAGS syndrome is inherited in the autosomal recessive mode. Here, we report a new case (14th up to date) of a patient with *ZNF699* gene mutation, whose symptoms and dysmorphic features were similar to those presented by Bertoli-Avella et al. In addition, we have analyzed the frequency of occurrence of particular symptoms in the patients described so far.

## 1. Introduction

The *ZNF699* gene (* 609571) is annotated in the National Center for Biotechnology Information (NCBI: http://www.ncbi.nlm.nih.gov/gquery/gquery.fcgi, accessed on 1 December 2021) as the human hang ortholog. It encodes a large nuclear zinc finger protein, suggesting a role in nucleic acid binding. There were two studies exploring the associations between variants in *ZNF699* and alcohol dependence [1,2]. 

Bertoli-Avella et al. in 2021 reported 13 patients with pathogenic homozygous variants in the *ZNF699* gene, improving a genotype-phenotype correlation in autosomal recessive mode of inheritance [3]. The phenotype is named DEGCAGS syndrome (OMIM 619488) and stands for Developmental Delay with Gastrointestinal, Cardiovascular, Genitourinary, and Skeletal Abnormalities [4]. 

In this paper, we present another patient with a mutation in the *ZNF699* gene, the 14th reported case, whose symptoms correspond to patients previously described by Bertoli-Avella et al.

## 2. Clinical Report

The patient is a one-year-old female, born from second pregnancy at 35 weeks gestation via cesarean section due to premature labor from healthy and unrelated parents. The first pregnancy was complicated by intrauterine fetal death at 40 weeks gestation, the pathomorphological section did not establish the cause of death, possibly an intrauterine infection. 

The birth weight was 1890 g (5 c), Apgar score was 7/7/8 points in the 1, 5 and 10 min of life. The pregnancy was complicated with polyhydroamniosis, single umbilical artery, and fetal ileus diagnosed prenatally. Aortic coarctation and shortened fetal long bones were suspected. In the 23rd week of pregnancy, amniocentesis was performed, and the fetal karyotype was normal (46, XX).

Shortly after birth, the patient required respiratory resuscitation, facial CPAP, and on the first day of life, was transferred to Newborn Intensive Care Unit (NICU) due to the severe general condition.

**Figure 1:**
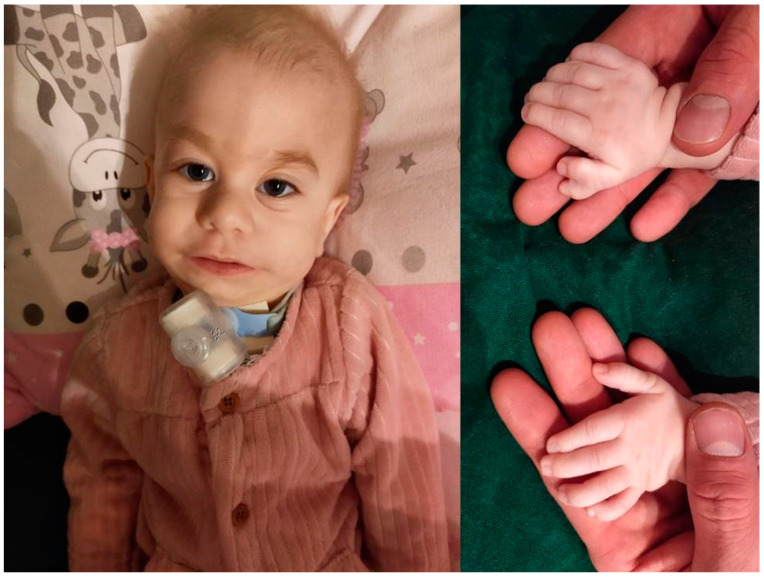
Dysmorphic features of the reported patient.

The infant was noticed to have dysmorphic features such as: a Saddle nose, low-set ears, retrognathia with micrognathia, suspicion of bilateral atresia of the external auditory canals, doubling of the right thumb, tendency to pinch five, overlapping second, fourth and fifth fingers on the middle finger, buffalo hump, livedo reticularis of the skin, and a sacral dimple (Figure 1).

In echocardiography, the aortic arch and isthmus were described as correct, no congenital heart defects were found.

The patient had a laparotomy with a massive short bowel resection (due to a multi-level obstruction of the jejunum-intestinal atresia) with one anastomosis on day 5 of life.

The patient underwent two more laparotomies due to digestive tract passage disorders, during which abdominal adhesions were removed and the anastomosis was resected due to its narrowing. Due to feeding intolerance, total parental nutrition was required all the time, the patient received trophic nutrition through a naso-intestinal tube.

Her first genetic consultation was at the age of 5.5 months. At that time, her growth parameters were below the third percentile for age and gender, weight was 4 kg, length 60 cm, and head circumference 38 cm. Dysmorphic features were noticed, as mentioned above. Developmentally, the girl had a global motor delay, central hypotonia with normal deep tendon reflexes. 

Due to the suspicion of atresia of the external auditory canals, the child had an ear-nose-throat evaluation which confirmed left side atresia of the left canal, the right was one was very narrow. 

Computer tomography of the brain showed widening of the ventricular system and enlarged post-cerebral fluid space as in atrophy. In the ultrasonography of the brain, higher echogenicity of periventricular white matter was found. In the ultrasonography of the abdomen, adrenal hyperplasia was described. Additionally, an atypical course of large vessels in the epigastric region, the aorta ran in sections on the right side, crossed by the inferior vena cava on the left side, in the lower sections, the course of the vessels was typical.

Due to severe infections (SARS-CoV-2 and three bacterial sepsis), the girl was hospitalized in the ICU. During one of the ICU stays, a tracheostomy was developed due to respiratory failure.

The following changes were found in the laboratory tests: Anemia, moderately elevated creatinine levels, constantly elevated ferritin levels (1100–1400 ng/mL, norm to 327 ng/mL), neuron-specific enolase was increased to 22.6 ng/mL (norm to 16.4 ng/mL). Other laboratory investigations were unremarkable (except for the moments when the child was in the ICU treated for infections): Newborn screening, liver function, ammonia, serum lactic acid, amino acid profile (GC/MS), CK, lipid panel. Her chromosomal karyotype was normal female 46, XX (amniocentesis), and array-CGH did not reveal any copy number changes.

## 3. Genetic Studies

### Whole Exome Sequencing (WES)

The DNA of the proband and her parents was isolated from peripheral blood lymphocytes, all according to standard protocols. For the proband, WES was performed using Human Core Exome Kit (Twist Bioscience, South San Francisco, CA, USA), according to manufacturer’s instruction. Enriched library was paired and end sequenced (2 × 100 bp) on a NovaSeq 6000 platform (Illumina, San Diego, CA, USA). Bioinformatic analysis of raw WES data and variants prioritization were performed as previously described [5]. Reads were aligned to the hg38 reference genome sequence and visualized using the Integrative Genomics Viewer [6]. 

**Figure 2:**
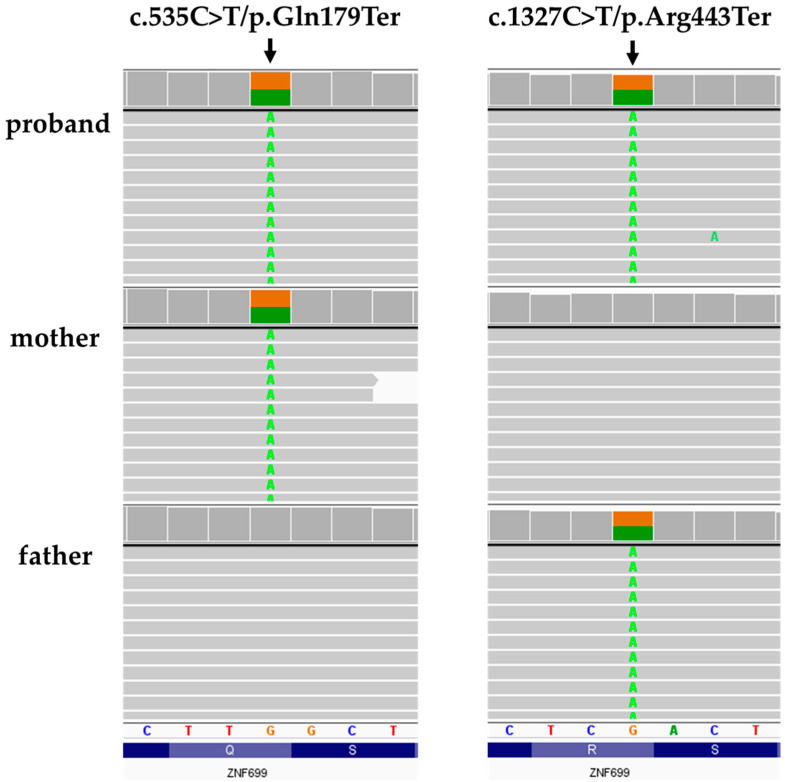
Family study results. Integrative Genomic Viewer screenshots are presented.

After the first tier of analysis, two nonsense variants in compound heterozygote state in *ZNF699* gene (NM_198535.3) were prioritized for further investigation: (hg 38, chr19:g.009296869-G>A; c.535C>T/p.Gln179Ter) and (hg38, chr19:g.009296077-G>A; c.1327C>T/p.Arg443Ter). According to gnomAD dataset (v.3.1.2) [7] the population frequency of p.Gln179Ter variant was zero, while for p.Arg443Ter was 0.00006515 (with no homozygotes reported). Both variants have zero frequency in in-house datasets of >11,000 WES of Polish individuals. Identified *ZNF699* p.Gln179Ter and p.Arg443Ter variants cause a premature occurrence of a stop codon and are responsible for shortened protein (the full length protein is 643 residues). Selected variants were further validated in the proband and studied in her parents by amplicon deep sequencing performed using the Nextera XT Kit (Illumina) and sequenced on a HiSeq 1500 (Illumina). Testing of the parents showed that the variant p.Gln179Ter was inherited from the mother, while p.Arg443Ter was inherited from the father (Figure 2), which is consistent with in trans variants transmission in the autosomal recessive mode of inheritance. 

The parents signed a written informed consent form for the genotyping and consented to the publishing of all the data generated. The study received the approval of the Bioethics Committee of Wroclaw Medical University (code: KB-430/2018; date of approval: 23 July 2018).

## 4. Discussion

The *ZNF699* gene (Chr. 19p13.2) is annotated in the NCBI as the human hang ortholog discovered by Scholz et al. in 2005 in Drosophila melanogaster and is required for normal development of ethanol tolerance. It encodes a large nuclear zinc finger protein, suggesting a role in nucleic acid binding, but still little is known about the function of this gene. 

Until 2021, *ZNF699* was not associated with any human disease. Bertoli-Avella et al. (2021) reported 13 patients from 12 unrelated consanguineous families of Arab descent with homozygous loss-of-function (LoF) variants in the *ZNF699* gene. In those cases, five different homozygous frameshift variants due to deletions or insertions were identified. All of them were predicted to result in premature termination. 

In our patient, we observe a configuration of a compound heterozygous due to substitutions in both alleles. Both variants in the *ZNF699* gene cause premature termination (p.Arg443Ter and p.Gln179Ter), which largely indicates their pathogenicity. These nonsense variants were not reported earlier in the literature.

All patients, including our case, presented a malformation syndrome with severe neurodevelopmental delay and the following main symptoms: Dysmorphic facial features, aberrations of the gastrointestinal, cardiovascular, genitourinary and skeletal system. In OMIM, the phenotype is called DEGCAGS syndrome (* 619488) and stands for Developmental Delay with Gastrointestinal, Cardiovascular, Genitourinary, and Skeletal Abnormalities.

We analyzed and summarized the symptoms of the disease in 14 patients. Less than half of the pregnancies (6/14) were complicated by at least one of the following: Intrauterine growth retardation (5/14), polyhydramnios (5/14), single umbilical artery (2/14), and premature birth (5/14). After birth, some patients needed respiratory support (CPAP, intubation). All patients had dysmorphic features, which included: An abnormal facial shape (9/14), microcephaly (6/14), long eyelashes (4/14), abnormal eyebrows (thick or unibrow, 4/14), nose abnormalities (prominent nasal bridge, upturned nose, short nose, 5/14) and other which included: Retrognathia, micrognathia, hypertelorism, smooth philtrum, macrotia, low set ears, low hairline, microphthalmia, ptosis, hypopigmentation of lashes and hair. 

Gastrointestinal abnormalities were significant in this group of patients. Intestinal atresia was observed in nine patients and was a reason for multiply operations. There were also feeding difficulties (4/14), and patients required nasogastric tube feeding. 

Skeletal anomalies included syndactyly (7/14), polydactyly (2/14), brachydactyly, absent thumbs, talipes equinovarus, and genu valgum.

In the analyzed group, the cardiovascular abnormalities seemed to be a smaller problem than gastrointestinal abnormalities and they concerned: Atrial septal defect (2/14), pulmonic stenosis (2/14) and individual cases of persistent left superior vena cava, ventricular septal defect, dysplastic pulmonary valve, patent formanem ovale, and patent ductus arteriosus. According to the available data, none of the patients required an intervention due to these features.

Renal hypoplasia (3/14), cryptorchidism (3/14), chronic kidney disease (2/14), and ambiguous (2/14), hypospadias (1/14), and chordee (1/14) were the observed symptoms from the genitourinary system.

In addition to the symptoms that are included in the acronym, we noticed the repetition of some symptoms. Eight patients had a failure to thrive, which may be primary or secondary to symptoms from the gastrointestinal or cardiovascular systems. Muscular hypotonia was present in seven patients. Five patients had recurrent infections, of those, two had confirmed immunodeficiency. Sensorineural hearing impairment was observed in four patients. In the respiratory tract, abnormalities concerned laryngomalacia, tracheomalacia, and bronchomalacia. 

In the laboratory findings, the most frequently reported deviation was in the complete blood count (8/14), two patients had pancytopenia, and six patients had anemia.

Analyzing the patients with a mutation(s) in the *ZNF699* gene, it is difficult to list specific symptoms that are characteristic and repetitive. Dysmorphic features (coarse facial, thick eyebrows, nose abnormalities, syndactyly), intestinal atresia that requires operation in the first days of life, and congenital heart defects (in general) seem to be the specific major symptoms that could be helpful to suspect the DEGCAGS syndrome in the early period of life.

## 5. Conclusions

Mutations causing premature termination in the *ZNF699* gene are responsible for new variable phenotype with characteristic dysmorphic facial features and significant structural defects, especially from the gastrointestinal, cardiovascular, and skeletal systems, and less specific from the genitourinary systems, accompanied by global developmental delay and failure to thrive.

## Data Availability

The data that support the findings of this study are available on request from the corresponding author [MR]. The data are not publicly available due to ethical restriction (data contain information that could compromise the privacy of research participants).
